# Reviving ghost alleles: Genetically admixed coyotes along the American Gulf Coast are critical for saving the endangered red wolf

**DOI:** 10.1126/sciadv.abn7731

**Published:** 2022-06-29

**Authors:** Bridgett M. vonHoldt, Joseph W. Hinton, Amy C. Shutt, Sean M. Murphy, Melissa L. Karlin, Jennifer R. Adams, Lisette P. Waits, Kristin E. Brzeski

**Affiliations:** 1 Department of Ecology & Evolutionary Biology, Princeton University, Princeton, NJ, USA.; 2 Wolf Conservation Center, South Salem, NY, USA.; 3 The Canid Project, Baton Rouge, LA, USA.; 4 Forestry and Natural Resources, University of Kentucky, Lexington, KY, USA.; 5 Department of Physics and Environmental Science, St. Mary’s University, San Antonio, TX, USA.; 6 Laboratory for Ecological, Evolutionary and Conservation Genetics, Department of Fish and Wildlife Sciences, University of Idaho, Moscow, ID, USA.; 7 College of Forest Resources and Environment Science, Michigan Technological University, Houghton, MI, USA.

## Abstract

The last known red wolves were captured in southwestern Louisiana and eastern Texas in 1980 to establish a captive breeding population. Before their extirpation, gene flow with coyotes resulted in the persistence of endangered red wolf genetic variation in local coyote populations. We assessed genomic ancestry and morphology of coyotes in southwestern Louisiana. We detected that 38 to 62% of the coyote genomes contained red wolf ancestry acquired in the past 30 years and have an admixture profile similar to that of the canids captured before the extirpation of red wolves. We further documented a positive correlation between ancestry and weight. Our findings highlight the importance of hybrids and admixed genomes as a reservoir of endangered species ancestry for innovative conservation efforts. Together, this work presents an unprecedented system that conservation can leverage to enrich the recovery program of an endangered species.

## Teaser

Coyotes along the American Gulf Coast carry genetic variation critical for the survival of the endangered red wolf species.

## INTRODUCTION

The conservation of hybrids remains a contentious and pressing issue in conservation biology (*1*). As human activities such as anthropogenic mortality, habitat degradation, and translocations of organisms promote increased incidents of species hybridization and introgression (*2*–*4*), increased interest for a web-of-life framework has been considered when developing conservation strategies for imperiled species (*5*). For example, allowing for some limited level of gene flow between species may facilitate genetic rescue for small, inbred populations (*6*) by countering the negative consequences of small effective population sizes with the positive consequences of novel allelic combinations (*7*, *8*). Further, such genetic exchange can promote rapid evolutionary innovation and adaptation, particularly under a changing climate (*3*, *8*, *9*), which may be considered an untapped mechanism of conservation and preservation of genetic variation. However, the policy for the management of hybrids and admixed individuals is unclear with hybrids rarely offered legal protections, partly because of the difficulty of classifying and measuring the impact of hybrids on parental species and environments (*10*). Yet, admixed genomes are a proven reservoir of putatively unique genetic and phenotypic combinations upon which natural selection could act (*3*).

Genomic research can identify signatures of past genetic exchange (i.e., ghosts of introgression) in admixed genomes (*11*). Genetic traits once thought extinct can be rediscovered and potentially revived when innovative conservation practices are considered. While traditional practices remain critical for species persistence, new genomic technologies paired with extreme reproductive assistance, such as cloning and biobanking, can expand the frontiers in conservation biology and hold new promises for species on the brink of extinction (*12*–*15*). Conservation practitioners are now supported with unprecedented technologies to construct clones or specific hybrid individuals that contain edited genomes that resurrect ghost variants and restore historic genetic variation. These pioneering methods create a space where admixed individuals play an important role in species conservation as critical reservoirs of ghost genetic variation.

Here, we provide a timely study pertinent to the red wolf (*Canis rufus*), a critically endangered species endemic to the southeastern United States, and coyote (*Canis latrans*), a species ubiquitous across North America. The survival of the red wolf could benefit from genomic technologies to bolster genetic variation as all extant red wolves are descended from the 14 founders, which has severe demographic and genetic consequences (*16*). Red wolves and coyotes have hybridized both historically and contemporarily (*17*–*21*). Most notably, during the mid-20th century, the last known red wolf populations along the Mississippi River Basin were extirpated, and the remaining wolves along coastal regions of eastern Texas and southwestern Louisiana (hereafter “SWLA”) began hybridizing with coyotes colonizing the region as wolf populations declined (*17*, *22*, *23*). Consequently, the U.S. Fish and Wildlife Service (USFWS) listed the red wolf as endangered and removed the last known individuals from the wild by 1980 to establish a captive breeding program as part of their Species Survival Plan (SSP) (*24*, *25*). The selection criterion was initially based on morphology, behavior, and health of captured canids, as the canonical red wolf phenotype was expected to be larger in size and proportion than hybrids or coyotes. However, to date, there has been no quantitative study to integrate morphology with genomic ancestry.

Despite the disappearance of the red wolf, reports of wolf-like canids in rural regions of coastal southeastern Texas and SWLA accumulated over the subsequent decades (*26*–*28*). Two recent independent studies substantiated these reports when red wolf ancestry was discovered in coyote populations occurring in southeastern Texas and SWLA (*27*, *28*). Further research has demonstrated that this Gulf Coast region likely represents a focal region of red wolf ancestry that has persisted since red wolf extirpation in the 1970s (*21*, *29*). Although these studies lacked associated morphology, it is reasonable to hypothesize that higher red wolf ancestry resulted in the large-bodied *Canis* documented in SWLA (*26*). A previous study identified introgression of putatively functional variation in the admixed genomes of canids of northeastern United States (*30*). Thus, given the variable phenotype and genomic ancestry observed across southeastern canids, we hypothesize a correlation with species-specific morphometrics, as measured by body size. These introgressed coyotes along the Gulf Coast states could represent a unique reservoir of previously lost red wolf ancestry, which has persisted in coyote genomes and could be critical for combating inbreeding in the genetically limited extant captive red wolf population. The integration of morphology and genome ancestry would present a uniquely powerful tool for prioritizing the selection of individuals to boost long-term health of the critically endangered red wolf species.

Here, we integrate genomic ancestry and morphology of coyotes living along the American Gulf Coast. We accomplished this by assessing red wolf ancestry in coyote populations along coastal SWLA where red wolf and coyote hybridization occurred (*17*, *28*, *31*). By capturing coyotes in these admixed populations, we acquired both genomic and morphologic data to identify the quantitative thresholds by which one could prioritize animals for potential use in ongoing red wolf recovery efforts based on individual ancestry proportions combined with phenotypic traits such as body size. Although it is known that hybrids are intermediate in size to red wolves and coyotes (*17*, *26*, *32*), the correlation of body size and ancestry is not well documented. Therefore, we investigated the effects of autosomal and X-linked red wolf ancestry on coyote body size. We then consider landscape characteristics that likely supported high retention of red wolf ancestry in coyote populations without management, revealing land cover where red wolf ancestry is most resilient. We suggest that hybrids are critical for defining what constitutes a red wolf, and admixed genomes will be pivotal in aiding red wolf conservation.

## RESULTS

### Capture and collaring of Louisiana coyotes

**Fig. 1.**
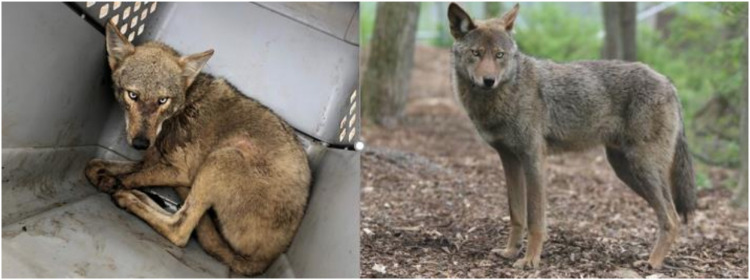
Comparison of coyote and red wolf. Coyote CL12928 (left; Photo by Joseph Hinton) captured in SWLA compared with a captive red wolf at the Wolf Conservation Center in New York (right; Photo by Maggie Howell).

We captured and radio-collared 26 coyotes (9 females and 17 males) from Cameron, Jefferson Davis, and Calcasieu parishes of SWLA between 7 February and 6 May 2021. We collected a combination of blood and ear tissue from radio-collared coyotes following the approved Institutional Animal Care and Use Committee (IACUC) protocol at the Michigan Technological University (no. 1677987-2). We opportunistically sampled ear tissue from seven road-killed coyotes (one female and six unknown) from Cameron parish in SWLA and a male SWLA coyote in a wildlife rehabilitation facility (East Baton Rouge and Iberville parishes). Coyotes in SWLA had a general appearance intermediate that of western coyotes and red wolves of North Carolina (Fig. 1) (*26*).

### Louisiana coyotes have genetic signals of reference coyotes and red wolves

**Fig. 2.**
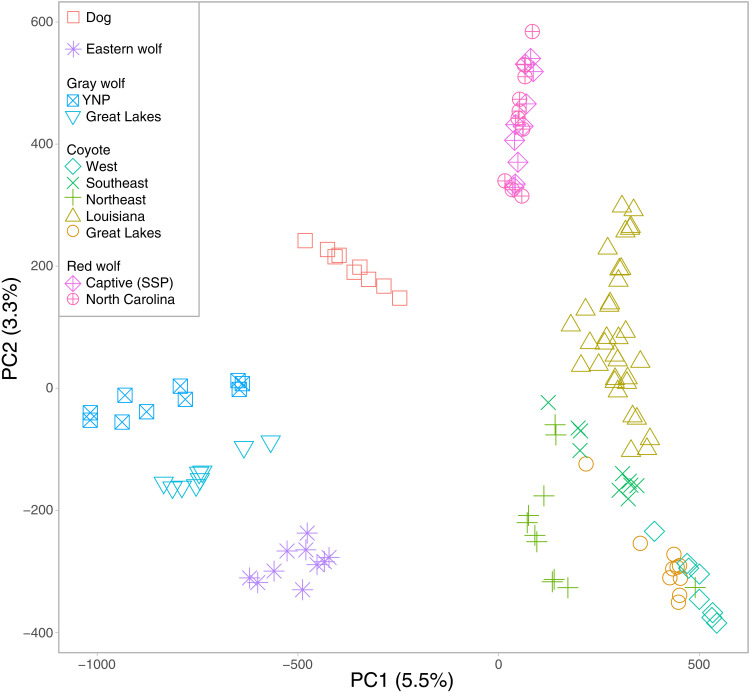
A PCA of 130 canids genotyped at 41,309 SNP loci. The percent of variation explained for each axis is provided in the parentheses. YNP, Yellowstone National Park.

Because of the high variability in the phenotype of SWLA coyotes (*26*), we obtained restriction site–associated DNA sequence (RADseq) data from 44 samples representing 34 unique coyotes from Louisiana and 10 red wolves from the North Carolina Nonessential Experimental Population (NCNEP) (table S1) (*33*). NCNEP red wolves were included for comparison given that they have experienced minimal introgressions from coyotes since reintroduction and could be genetically similar to canids along the Gulf Coast. We merged the genome-wide single-nucleotide polymorphism (SNP) genotype data with publicly available data from an additional 88 canids that represented several distinct reference lineages: 10 domestic dogs, 39 coyotes, 19 gray wolves, 10 eastern wolves, and 10 captive red wolves from the SSP population (table S1) (*21*, *27*, *34*). After extensive data filtering, we retained 130 canids and 59,788 SNP loci out of a total of 199,888 cataloged variants. Additional filtering for linkage and Hardy-Weinberg equilibrium (HWE) deviations established a subset of 41,309 SNP loci that we designated as statistically neutral and unlinked. A principal components analysis (PCA) revealed the expected clustering of each canid reference lineage, while the NCNEP red wolves clustered tightly with the SSP reference red wolves and the Louisiana coyotes spanned two principal component 2 (PC2) clusters of red wolves and coyotes (Fig. 2). Given the lack of variation among the NCNEP red wolves, they were included with the SSP reference red wolves for downstream analyses.

### Coyotes of SWLA carry high red wolf ancestry with recent admixture dates

**Fig. 3.**
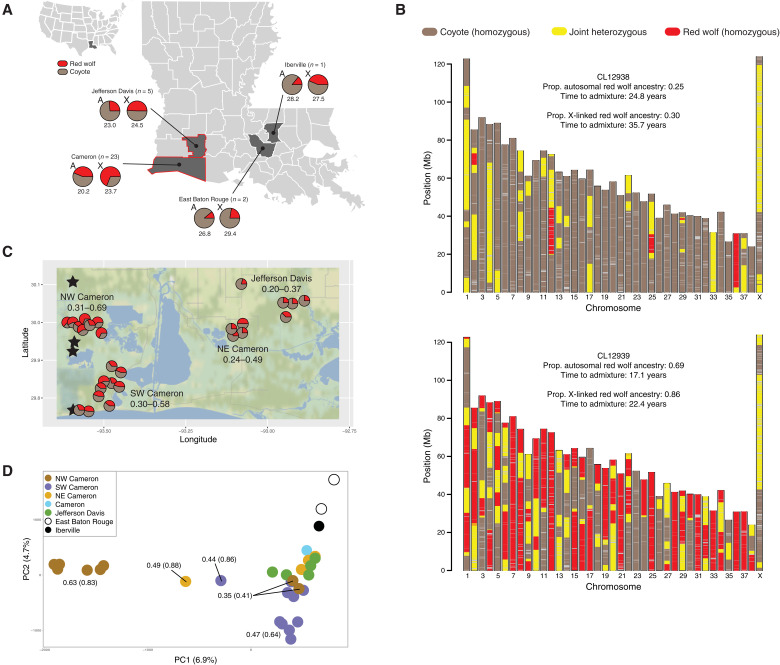
Genomic ancestry of 31 Louisiana coyotes. (**A**) Genomic ancestry proportions inferred from 59,788 SNP loci across autosomes (A) and the X chromosome (X) with respect to two reference lineages: 39 reference coyotes and 10 reference red wolves from the captive SSP population. The average timing of admixture is provided (in years) below the pie charts. The Louisiana parishes outlined in red are enlarged in (C). (**B**) Chromosomal plots of ancestry fragments for two Louisiana coyotes. Fragments across each of the 38 autosomes and X chromosome are color-coded with respect to ancestry state. These two individuals were selected to display the lowest and oldest or highest and most recent red wolf proportions (CL12938 and CL12939, respectively). Sample identity, ancestry proportions, and admixture timing estimates are provided above each plot. (**C**) Depiction of (A) (red outlined parishes) with higher-resolution spatial details of autosomal proportions of red wolf ancestry (min-max) for each coyote with latitude/longitude data (see table S1). Stars indicate the geographic locations where four red wolf SSP founders originated. Sample sizes (*n*) and parish names are provided. (**D**) The PCA of 31 Louisiana coyotes revealed PC1 as an axis of red wolf ancestry (average autosomal red wolf proportions are provided for each color symbol with X chromosome proportions in parenthesis in the PCA). The percent of variance explained by each component is provided on each axis. NE, northeast; NW, northwest; Prop., proportion; SW, southwest.

To investigate the degree and geographic extent to which these coyotes may be a reservoir for lost red wolf genetic variation, we inferred red wolf ancestry proportions for 31 Louisiana coyotes across 59,788 SNP loci. We found that these individuals displayed variable red wolf ancestry proportions across the autosomes (means ± SD = 0.38 ± 0.2) and X chromosome (0.62 ± 0.3) (Table 1). Given our limited geographic access for sample collection, we have an enrichment of genetic representation within Cameron Parish; however, concordant with past findings, this parish also contained the highest red wolf ancestry proportions (min-max: autosomes = 0.18 to 0.69, X chromosome = 0.18 to 1.0) with the most recent estimated admixture timing (autosomes = 20 years, X chromosome = 24 years) (Fig. 3A and Table 1) (*21*, *29*). The other three Louisiana parishes collectively analyzed (Jefferson Davis, Iberville, and East Baton Rouge) were significantly lower in average red wolf ancestry (autosomes = 0.21; *t* test, *P* = 2 × 10^−5^; X chromosome = 0.42, *P* = 0.0035) with older admixture timing (autosomes = 25 years, *P* = 0.002; X chromosome = 26 years, *P* = 0.2554). We visualized the location of ancestry blocks across the chromosomes of a coyote with the lowest red wolf proportions (sample CL12938), alongside the coyote with the highest red wolf proportions (sample CL12939). We found that such fragments are frequently in the heterozygous state for the low red wolf content coyote, while the high red wolf content coyote’s genome carries a substantially higher frequency of homozygous red wolf blocks (Fig. 3B).

**Table 1.** Proportion and timing (in years) of red wolf genomic ancestry for 31 coyotes captured and sampled in SWLA. Genomic ancestry was inferred across 59,788 SNPs genotyped in 31 coyotes from Louisiana with respect to 39 reference coyotes and 10 reference red wolves from the captive SSP population. Prop., proportion.

		**Prop. of red wolf ancestry**		**Timing of red wolf admixture**	
**Sample**	**Louisiana parish**	**Autosomal**	**X-linked**	**Autosomal**	**X-linked**
CL12923	Cameron	0.629	0.561	16.5	35.7
CL12924	Cameron	0.678	0.957	14.7	0.0
CL12926	Cameron	0.635	0.883	15.1	26.2
CL12927	Cameron	0.573	1.000	17.5	0.0
CL12928	Cameron	0.394	0.632	20.8	34.9
CL12929	Cameron	0.595	0.723	16.9	20.2
CL12930	Cameron	0.487	0.877	19.7	31.3
CL12931	Cameron	0.241	0.901	25.4	37.9
CL12932	Cameron	0.286	0.695	23.1	25.5
CL12933	Cameron	0.254	0.526	24.9	16.3
CL12935	Jefferson Davis	0.374	0.693	19.5	28.6
CL12936	Jefferson Davis	0.247	0.314	20.7	18.8
CL12937	Jefferson Davis	0.238	0.475	24.0	10.8
CL12938	Jefferson Davis	0.249	0.296	24.8	35.7
CL12939	Cameron	0.693	0.860	17.1	22.4
CL12940	Cameron	0.312	0.184	21.6	18.0
CL12973	Cameron	0.467	0.889	18.4	20.5
CL12974	Cameron	0.300	0.911	23.6	25.0
CL12975	Cameron	0.351	0.525	21.0	24.4
CL12976	Cameron	0.435	0.860	19.8	16.8
CL12977	Cameron	0.579	0.311	19.3	18.9
CL12978	Cameron	0.391	0.868	21.0	35.4
CL12979	Cameron	0.451	0.501	19.8	27.0
CL12980	Cameron	0.389	0.743	209.	31.8
CL12981	Jefferson Davis	0.207	0.691	26.1	28.5
CL12982	Cameron	0.446	0.777	19.1	19.5
CL12983	Cameron	0.312	0.447	22.6	38.3
CL13003	East Baton Rouge	0.098	0.336	26.5	24.2
CL13004	East Baton Rouge	0.124	0.110	27.2	34.5
CL13005	Iberville	0.141	0.437	28.2	27.5
CL13006	Calcasieu	0.182	0.249	26.6	18.1

We quantified the number of alleles private to the SWLA coyotes and not found in the reference groups included in this study, which included red wolf genomes representing both the SSP and the NCNEP genomic variation. We discovered that the SWLA coyotes carried 185 private alleles, five times as many as found in red wolves (*n* = 38) and comparable to other wild canids (gray wolves, *n* = 238; coyote, *n* = 483), and a significant number more than populations in decline or reproductively isolated (eastern wolves, *n* = 10; domestic dogs, *n* = 6) (fig. S1). This same trend was even more notable when we analyzed a subset of canids to compare to the 10 canids from the 1970s capture efforts, which carried 50 private alleles compared to the 576 identified in the contemporary SWLA coyotes, 50 in red wolves, and 2105 in coyotes (fig. S1).

### Regional discovery of land preserves that lack hunting has highest red wolf ancestry

Of particular interest are the 28 coyotes sampled from Cameron and Jefferson Davis Parishes (Fig. 3A). Land cover across the region changes considerably with increasing distance from the Gulf Coast shoreline that was composed of a complex mosaic of saline to intermediate marsh zones in Louisiana (*35*). Much of the landscape in which we sampled coyotes had limited hunting access. For example, all 26 coyotes that we captured and radio-collared were of healthy weight, and annual mortality appeared relatively low (vehicle collision, *N* = 2; predator control trapping, *N* = 1; capture myopathy, *N* = 1; unknown cause, *N* = 1). To note, our long-term goal is to establish a noninvasive assay to expand sampling and reduce stressful encounters for animals. Coyotes with the highest red wolf ancestry were sampled in northwestern Cameron Parish (autosomes = 0.56, X chromosome = 0.73) on a private ranch that prohibited hunting and trapping of wildlife, followed by southwestern Cameron Parish (autosomes = 0.41, X chromosome = 0.68) on Sabine National Wildlife Refuge and corporate oil holdings with limited public access, northeast Cameron Parish (autosomes = 0.32, X chromosome = 0.75) on Cameron Prairie National Wildlife Refuge and surrounding private lands that permitted hunting, and Jefferson Davis Parish (autosomes = 0.26, X chromosome = 0.49) on private land with active coyote control around its exotic hunting preserve (Fig. 3C). Chromosomal fragments of red wolf ancestry in the coyotes of northwestern Cameron Parish were the most recently acquired (autosomes = 17.5 years, X chromosome = 19.7 years) and again follow the same trend with older admixture time estimates with decreasing ancestry proportions (southwestern Cameron Parish: autosomes = 20.5, X chromosome = 25.8; northeast Cameron Parish: autosomes = 23.3, X chromosome = 27.7; Jefferson Davis Parish: autosomes = 23.0, X chromosome = 24.5).

We conducted a PCA of genotype data from the 31 Louisiana coyotes and found that PC1 was negatively correlated with the average autosomal red wolf ancestry for each geographic origin of the samples (*r* = −0.844) and, to a lesser degree, ancestry on the X chromosome (*r* = −0.517) (Fig. 3D). We also find the continued support that coyote populations represent a mosaic of individuals with tremendous interindividual variation in red wolf ancestry proportions, exemplified by the coyotes from northwestern Cameron Parish. Although this geographic cluster of samples contains individuals with the highest estimated red wolf ancestry, there are two with lower estimates and clusters with similar ancestry proportions on the PCA (Fig. 3D).

The average longest homozygous red wolf ancestry blocks were carried by coyotes in northwestern Cameron Parish (56.2 ± 49.4 Mb), relative to the other geographic clusters within the parish (northeast = 18.7 ± 16.9 Mb and southwest = 3.0 ± 2.2 Mb) and in the neighboring Jefferson Davis Parish (12.1 ± 6.7 Mb) (Table 2). This trend, however, is predominantly driven by three outlier individuals with extremely long homozygous red wolf ancestry blocks (87.2 to 122.6 Mb). The ratio of homozygous red wolf to coyote ancestry block sizes also revealed that coyotes in northwestern Cameron Parish had red wolf ancestry blocks 3.5 times longer than their homozygous coyote block sizes (56.2 and 16.2 Mb, respectively). Coyotes in northeastern Cameron Parish carried the next longest red wolf ancestry blocks, 1.6 times longer than homozygous coyote blocks (18.7 and 11.7 Mb), in addition to this region exhibiting older admixture time estimates relative to the northwestern Cameron region.

**Table 2.** Autosomal ancestry block sizes (in megabase) for 31 coyotes captured and sampled in SWLA. Average block sizes for each ancestry state per coyote. Joint is defined as the heterozygous ancestry state. NE, northeast; NW, northwest; SW, southwest.

**Sample**	**Louisiana parish**	**Coyote**	**Joint**	**Red wolf**
CL12923	Cameron (NW)	16.9	24.2	122.6
CL12924	Cameron (NW)	9.0	25.7	122.6
CL12926	Cameron (NW)	8.7	3.9	47.6
CL12927	Cameron (NW)	14.9	7.8	16.0
CL12928	Cameron (NW)	36.7	3.1	4.0
CL12929	Cameron (NW)	9.1	26.6	87.2
CL12930	Cameron (NE)	18.6	7.2	5.0
CL12931	Cameron (NE)	23.5	0.7	3.1
CL12932	Cameron (NE)	4.2	9.8	34.2
CL12933	Cameron (NE)	0.5	0.3	32.4
CL12935	Jefferson Davis	13.6	0.7	11.4
CL12936	Jefferson Davis	6.7	8.2	23.7
CL12937	Jefferson Davis	2.3	1.5	9.1
CL12938	Jefferson Davis	30.8	46.3	8.3
CL12939	Cameron (NW)	9.3	2.8	46.3
CL12940	Cameron (NW)	24.9	0.4	3.6
CL12973	Cameron (SW)	13.3	20.1	5.9
CL12974	Cameron (SW)	20.9	1.2	2.5
CL12975	Cameron (SW)	28.8	30.8	0.7
CL12976	Cameron (SW)	5.1	1.1	2.8
CL12977	Cameron (SW)	32.1	4.4	0.6
CL12978	Cameron (SW)	10.7	8.0	5.1
CL12979	Cameron (SW)	2.2	31.8	1.6
CL12980	Cameron (SW)	33.6	0.6	3.1
CL12981	Jefferson Davis	7.6	16.2	7.8
CL12982	Cameron (SW)	10.1	18.8	6.9
CL12983	Cameron (SW)	31.4	0.2	1.0
CL13003	East Baton Rouge	13.9	11.4	6.0
CL13004	East Baton Rouge	13.9	1.6	9.4
CL13005	Iberville	15.4	13.0	10.4
CL13006	Calcasieu	26.4	1.9	2.9

### Morphology and red wolf ancestry

**Fig. 4.**
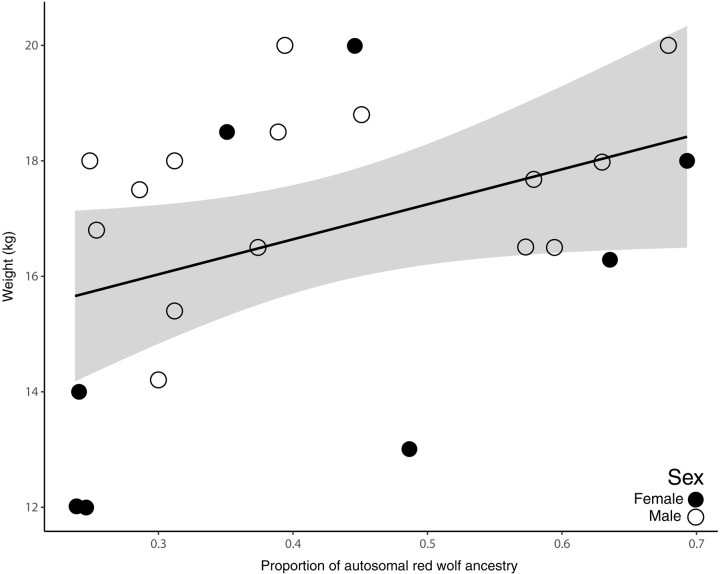
Body size and autosomal red wolf ancestry. Association between autosomal red wolf ancestry proportions and body size, as measured as weight (kilograms), for 24 live-captured Louisiana coyotes with ancestry estimates. The solid line is the fitted regression line, and the gray-shaded area represents SE of the linear model (β = 7.42, SE = 3.22, *R*^2^ = 0.25).

We correlated body size of coyotes with red wolf ancestry estimates and found that coyotes with higher red wolf autosomal ancestry were, on average, heavier animals (Fig. 4 and tables S3 and S4). Coyote body mass was positively correlated with autosomal red wolf ancestry (β = 7.42, SE = 3.22, and 95% confidence intervals = 1.39 to 13.44), where the top-ranked model [body mass Akaike Information Criterion (*AIC*) = 0.82] also included sex (β = 2.1, SE = 0.85, and 95% confidence intervals = 0.45 to 3.78). X-linked red wolf ancestry was negatively associated with weight, albeit this was not a strong or significant trend (β = −0.01, SE = 2.11, and 95% confidence intervals = −4.10 to 4.42).

### Signatures of red wolf genetic variation in SWLA coyotes are similar to Texas canids from the 1970s

**Fig. 5.**
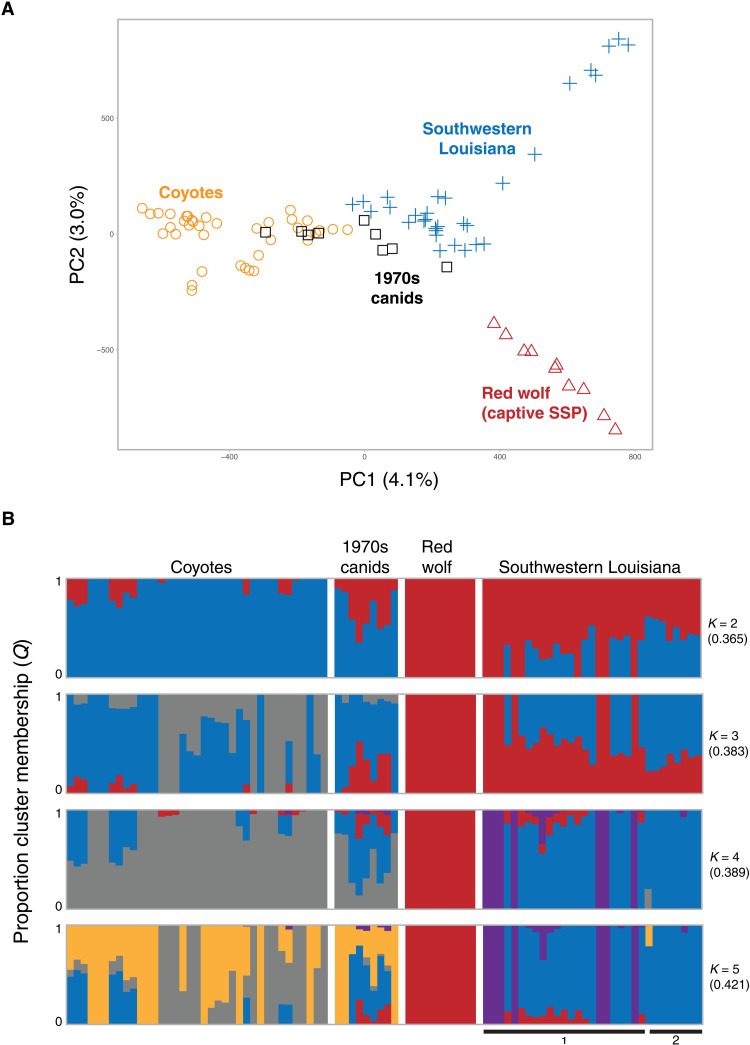
Genetic variation patterns of SWLA coyotes with respect to Texas canids captured during the 1970s across 45,994 unlinked and neutral SNPs. (**A**) A PCA of 87 canids, and (**B**) maximum likelihood cluster analysis for the best-fit partition (*K* = 2) and additional subsequent partitions (*K* = 3 to 5). The cross-validation (cv) error per partition is given in the parentheses. The solid bar below the graph indicates the Parish of sample origins in Louisiana (1, Cameron Parish; 2, East Baton Rouge, Iberville, and Jefferson Davis Parishes).

We included genotype data from 10 Texas canids sampled during the 1974–1980 red wolf founder capture efforts but not included in the final breeding program (Table 3). We annotated and genotyped 47 reference canids (coyote = 37 and red wolf = 10), 9 canids from 1970s, and 31 coyotes from SWLA for 45,994 SNPs after filtering for minor allele frequency (MAF), missingness, genotype correlation, and deviations from HWE. We found that several SWLA coyotes cluster in PC space proximal to canids from the 1970s capture events (Fig. 5A), with a maximum likelihood model–based approach discovering that coyotes from Cameron Parish have similar membership proportions to red wolf cluster as the 1970s canids when the data is assessed at four genetic partitions (*K*) (6 and 9.2%, respectively), relative to the other samples from SWLA parishes (<1%) (Fig. 5B). Such membership proportion trends hold for other partitions (*K* = 3: 23.6, 64.1, and 32.6%; *K* = 5: 8.3, 5.8, and <1%).

**Table 3.** Sample information for 10 Texas canids from the 1970s red wolf capture efforts. Red wolf ancestry proportions are from previously published genome analyses (*21*).

**Sample ID** **(county in Texas)**	**Red wolf ancestry** **proportions**	**Collection date**
70-TX-01 (Webb)	0.02	22 March 1976
70-TX-02 (Webb)	0.02	23 March 1976
70-TX-03 (Jefferson)*	0.11	24 January 1976
70-TX-04 (Jefferson)*	0.52	25 January 1976
70-TX-05 (Harris)*	0.19	4 January 1976
70-TX-06 (Montague)	0.02	21 November 1975
70-TX-07 (Brazoria)*	0.03	2 February 1975
70-TX-08 (Liberty)*	0.27	31 July 1975
70-TX-09 (Brazoria)*	0.29	7 May 1975
70-TX-10 (Webb)	0.02	23 March 1976

*Counties geographically adjacent to or are actual red wolf SSP founder source locations.

## DISCUSSION

Coastal SWLA is a particularly important locality for assessing red wolf–coyote hybridization as it was (i) the last known area occupied by red wolves before their extirpation from the wild in 1980 (*17*, *31*) and (ii) one of the first regions in the eastern United States to be colonized by coyotes (*36*). We observed a range of red wolf autosomal ancestry (10 to 69%) in coyotes along coastal SWLA estimated to have occurred in the past 30 years. Coyotes with the longest and oldest contiguous chromosomal fragments of red wolf ancestry were found in the remote and isolated wetlands of Cameron Parish. In particular, coyotes with the greatest red wolf ancestry were on the FR Ranch of the Moore-Odom Wildlife Foundation, a property that does not permit hunting and trapping of wildlife. Our findings suggest that areas with reduced lethal management allow for the persistence of red wolf ancestry in coyote populations. Given that anthropogenic mortality of red wolves promotes wolf-coyote hybridization (*37*–*39*), we were not surprised that red wolf ancestry was greatest in coyotes residing in such isolated areas that afforded reduced exposure to lethal control.

We found a positive correlation between coyote body size and autosomal red wolf ancestry and observed no coyotes with the majority (>50%) of autosomal red wolf ancestry that weighed less than 15.5 kg. In North Carolina, body size can be a reliable predictor of home range size and thus species identity, with larger-bodied wolves often holding larger average home ranges than coyotes (55 and 30 km^2^, respectively) (*39*). Further, body size combined with space use contributes to red wolf and coyote assortative mating (*39*). The X chromosome has a more complex mode and history upon which natural selection can act. We find an enrichment of higher red wolf ancestry on this sex chromosome and suspect that, given more data, there are possibly sex-based differences in demography, life history, and fitness. Albeit a small sample size, our findings indicate that the persistence of large-bodied coyotes in SWLA is due to the inheritance of autosomal red wolf ancestry. Phenotypic characters such as body size and pelage color are helpful to identify hybrids and introgressed coyotes (*32*, *36*). For example, (*32*) reported that only red wolf pups who had not achieved adult-like body sizes, rather than juvenile and adult red wolves, were confused for hybrids. They noted that hybrids were more similar to coyotes in body size and that no specific morphological character, except intermediate measurements and appearance, was used to differentiate hybrids from coyotes. Similarly, coyotes in SWLA that were highly introgressed with red wolf ancestry were phenotypically often more similar to coyotes than to red wolves, suggesting that genomic analyses using various genes are necessary to evaluate the extent of wolf introgression in the region’s coyote population.

Population surveys that use genomic analyses will be critical to characterize the dynamics of the red wolf–coyote hybrid zone in the southeastern United States. The interindividual variation observed here highlights the need for rapid genome-level scans of populations critical for conservation practitioners and recovery programs. Now, it appears that remnants of the hybrid zone are confined to isolated habitats along the Gulf Coast that are considerably isolated from adjacent human activities and southeastern coyote populations. Our findings indicate that red wolf ancestry blocks entered into these coyotes’ genomes as recently as 20 to 30 years ago in SWLA. Coincidently, the USFWS Red Wolf Recovery Program resurveyed the region for red wolves during the early 1990s and our findings provide evidence that red wolf-like canids may have persisted into the early 1990s.

### Importance of the admixture zone

Coastal SWLA represents a complex admixture zone and reservoir of presumed lost red wolf genomic variation as it persists in admixed coyote genomes. Ghost genetic variation is a crucial signature of past gene flow and has been identified as a mechanism to retain the endangered genomic variation of the ancestral red wolf population that was believed to be lost from the wild. Here, we found that the coyotes of SWLA, persisting in the red wolf ancestral range, carry an incredible number of alleles not found in any other North American canid analyzed. This rediscovered genomic diversity may hold the key to distance the red wolf species from the brink of extinction.

Further, given the enrichment of red wolf variation now documented in this region, we suggest that SWLA should be prioritized as a potential site for a future red wolf reintroduction. This natural occurrence of endangered genetic variation provides a redundant conservation design, supporting the SSP red wolf breeding efforts while providing a redundant and independent effective population. Our study presents one of the first connections between the red wolf phenotype and genomic estimates of red wolf ancestry. Building upon previous work that supports red wolf–specific traits (*32*, *35*, *39*), we have documented a positive correlation between these traits and genomic ancestry in coyotes of the historic red wolf admixture zone (*17*).

Conservation practitioners are eager to implement innovative conservation strategies that incorporate functional genomic variation. Although we report the positive correlation between phenotype and ancestry proportions, it remains unclear which genomic regions are crucial for maintaining the red wolf phenotype. This challenge is driven by the historic distribution of red wolves across a diverse range of habitats, compounded by the rapid rate at which their historical landscape was permanently altered by European colonization and anthropogenic activity. We expect that, with further modeling of quantitative morphometrics and genomic variation, adaptive ancestry blocks can be linked to specific traits that are central to red wolf fitness, behavior, and ecology. Combined with ecological modeling as climate change will alter landscapes, this could be a powerful moment for integrating across several biological dimensions.

A final challenge presented in the admixture zone is species assignment. When a coyote is estimated to carry a predominant estimate of red wolf genomic ancestry proportion (i.e., >50%), we argue that such individuals are a crucial component for the persistence of the endangered red wolf. We have the tools to integrate ancestry estimates with ancestry block metrics (e.g., length and identity) to estimate the timing of which such events occurred. Our findings can provide a hopeful precedent for other conservation situations that face challenges due to introgression, such as the Przewalski’s horse (*40*) and European wild cats (*41*, *42*). We encourage conservation practitioners to go beyond species concepts and pioneer a vision that leverages admixture to provide endangered genomes with the best possible probability for survival in our rapidly changing world.

### Conservation strategies

Species recovery plans have traditionally been organized around the model-based population viability analysis (PVA) to develop measurable recovery criteria (*43*). Challenges to such PVA-centered structures have identified that such a method is not universally tractable for all listed species, is computationally data intensive, and is constrained to the model’s time frame (*43*, *44*). Recovery plans are now structured on a conservation biology framework (“The three R’s”) focused on the Endangered Species Act’s (ESA) requirements of geographic representation of the species, conservation of the relevant ecosystems for the species to be self-sustaining, and abatement of threats (*45*). This recent restructuring should result in the establishment of multiple large, genetically robust, self-sustaining populations across the species’ range and all ecological contexts.

As part of a recent effort for reevaluating red wolf recovery, the Association of Zoos and Aquariums’ American Red Wolf SAFE Program Action Plan (2019 to 2022) conducted a PVA and recommended that stakeholders work to ensure an ex situ population to support continued recovery efforts. Here, we defined substantial levels of red wolf genomic ancestry in coyotes of SWLA that are thriving on land where lethal management is not permitted. More than 50 years ago, the last of the wild red wolves were documented in this region prior to being declared extinct in the wild (*17*, *18*, *22*, *23*, *31*, *46*–*48*).

Given the high levels of red wolf ancestry in coyotes along coastal SWLA, we suggest that these coyote populations represent a potential for conservation redundancy of red wolf genes and the persistence of ancestral variation once thought to be extinct in the wild. As coastal SWLA is within the recent historic range of red wolves, including these populations in the red wolf’s three R’s recovery plan will promote the red wolves’ potential for adaptation, especially in a changing climate. Alongside PVA models, we suggest that conservation strategies include a mechanism to prioritize several aspects of admixed genomes (e.g., timing since admixture, percent content, and ancestry block length) and thus the red wolf genomic legacies. These ghost genomes have naturally persisted in isolated areas for several decades through means that are not yet well understood.

We do acknowledge the challenge for implementation of a strict genomic ancestry profile. For example, morphometrics will be crucial for understanding the influence of red wolf ancestry on canid adaptation to anthropogenic landscapes. This is especially important given that landscape changes across the red wolves historic range. Our findings of higher red wolf ancestry proportions in SWLA may also be explained, in part, by the history of clear-cutting and livestock operations initiated in the late 1800s (*49*). Was early coyote–red wolf hybridization (*18*, *46*) a possible mechanism by which these canids were able to survive in a rapidly fragmented and converted landscape? Exclusion of individuals from conservation protection that do not conform to a phenotypic standard of an endangered species may result in the major oversight or exclusion of critical genomic variation potentially useful for genomic rescue or local adaptation through targeted practices.

As technology continues to provide innovative methods, the Gulf Coast canids also represent a critical biobanking opportunity for when genome editing methods are applied to red wolves. These methods were recently developed as a therapeutic technique to replace targeted gene sequences through the DNA repair process or transiently modify RNA (*50*). The consideration of these pioneering methods is the new frontier of conservation science for endangered species in the era of anthropogenic-driven biodiversity loss and maladaptation due to rapidly changing climate and landscapes (*14*, *15*). We are at a pivotal moment where red wolves can be at the forefront to benefit from these developing conservation tools, and it is imperative to act quickly to preserve and harness red wolf ghost genomes now only present in Gulf Coast canids.

## MATERIALS AND METHODS

### Experimental design

#### Sample collection

From February to May 2021, we captured 26 coyotes using foothold traps with offset jaws (Minnesota Brand 550, Minnesota Trapline Products, Pennock, MN, USA). Once captured, animals were restrained with a catchpole, muzzle, and hobbles. When needed, we chemically immobilized animals with an intramuscular injection of ketamine HCl (1.3 mg/kg) and xylazine HCl (0.2 mg/kg) to inspect inside their mouths for injuries. We recorded sex, weight, and body measurements for all animals and estimated age by tooth wear (table S2) (*51*, *52*). We categorized animals ≥2 years as adults, 1 to 2 years old as juveniles, and less than 1 year old as pups. We collected 5 ml of whole blood in Longmire buffer from the cephalic veins of captured coyotes and opportunistically sampled ear tissue from road-killed coyotes. All coyotes were fitted with Lotek LiteTrack Iridium 360 GPS collars (Lotek, Newmarket, ON, Canada). Our capture and handling of animals followed the guidelines approved by the American Society of Mammalogists (2020) and were approved by the IACUC at the Michigan Technological University (no. 1677987-2). We plotted latitude and longitude for each sampled location using the qmplot function in the ggmap v3.0.0 R package (*53*).

#### DNA extraction

We collected high–molecular weight genomic DNA from whole blood or tissue from 36 coyotes sampled from Louisiana and 10 red wolves from North Carolina using the DNeasy Blood and Tissue Kit (QIAGEN) and followed the manufacturer’s protocol for mammals. We quantified DNA concentration using the Qubit 2.0 fluorometer system and subsequently standardized DNA to 5 ng/μl.

#### RADseq and bioinformatic processing

We prepared 46 (two samples were duplicated) genomic libraries for RADseq following a modified protocol (*54*). Briefly, we used the *Sbf1* restriction enzyme to digest genomic DNA and ligated a unique 8–base pair (bp) barcoded biotinylated adapter to the resulting fragments. The barcode allows us to pool equal amounts of each DNA sample followed by random shearing to 400 bp in a Covaris LE220. We used a Dynabeads M-280 streptavidin binding assay to enrich the pools for adapter-ligated fragments, followed by a size selection for fragments of 300 to 400 bp in size and purification using Agencourt AMPure XP magnetic beads. The libraries were then prepared for Illumina NovaSeq 2 × 150-nt sequencing at Princeton University’s Lewis-Sigler Genomics Institute Core Facility using the NEBNext Ultra II DNA Library Prep Kit.

We retained sequencing reads that contained the unique barcode and the remnant *SbfI* cut site. We processed read data in STACKS v2 to first demultiplex the pools using 2-bp mismatch for barcode rescue in the process_radtags module. We retained reads with a quality score ≥ 10 and removed polymerase chain reaction duplicates with the paired-end sequencing filtering option with the clone_filter module. Cleaned reads were then mapped to the dog genome CanFam3.1 assembly (*55*) using BWA-mem (*56*). We also filtered mapped reads for a minimum MAPQ of 20 and converted to bam format in Samtools v0.1.18 (*57*). We included RADseq data from 88 canids that were previously published (coyotes = 39, gray wolves = 19, eastern wolves = 10, and captive red wolves = 10) (table S1). The 88 publicly available canid samples were included as processed reads and mapped to the same reference genome assembly following these methods.

We completed SNP discovery using all samples to obtain a catalog of all polymorphic sites possible. We followed the recommended pipeline for the gstacks and populations modules in STACKS v2 after the data were mapped to a reference genome (*58*, *59*). However, we increased the minimum significance threshold in gstacks to require more stringent confidence needed to identify a polymorphic site using the marukilow model (flags --vt-alpha and --gt-alpha, *P* = 0.01). We reported all SNPs discovered per locus (opted against using the populations flag --write_single_snp) as ancestry inference is best with high-density data. We then used VCFtools v0.1.17 (*60*) to exclude singleton and private doubleton alleles, remove loci with more than 90% missing data across all samples, and remove individuals with more than 20% missing data (we excluded four samples; table S1). We filtered for a minimum of 3% MAF in PLINK v1.90b3i (*61*). For initial screening of the samples, we constructed a “statistically neutral and unlinked” dataset of SNPs by excluding sites within 50-SNP windows that exceeded genotype correlations of 0.5 (with the PLINK argument *--*indep-pairwise 50 5 0.5) and deviated from HWE with the argument --hwe 0.001. The PCA was completed in the program flashPCA (*62*).

#### Inclusion of 1970s canids from Texas

We included publicly available BAM files from 10 canid samples in the 1970s from Texas, previously mapped to the same reference genome assembly (*21*). Following the methods and thresholds detailed above, we annotated SNPs across 47 reference canids (37 coyotes and 10 red wolves), 10 canids captured during the 1970s, and the 31 coyotes from SWLA. Samples were excluded from downstream analyses if they contained at least 20% missing data.

### Statistical analysis

#### Inference of canid ancestry

We inferred local ancestry of 36 coyotes from Louisiana with possible red wolf ancestry with respect to two reference populations: coyotes and red wolves (defined in table S1). Following our past methods, briefly, we implemented a two-layer hidden Markov model in the program Efficient Local Ancestry Inference (ELAI) to infer local genomic ancestry proportions for the 59,788 SNP set (*63*). We used the following parameters: *-C* set to 2 and -*c* set to 10. As the precise nature of admixture is unknown, we analyzed four time points since admixture (-*mg*): 5, 10, 15, and 20 generations. We implemented ELAI three times serially for each -*mg* parameter value with 30 expectation-maximization (EM) steps and averaged results over all 12 independent analyses. ELAI returns a per-SNP allele dosage score, which estimates the most likely ancestry proportion. We assigned chromosomal positions with allele dosage between 0.8 and 1.8 as heterozygous and those with allele dosage >1.8 as homozygous.

#### Estimating the timing of admixture

We counted the number of ancestry block identity switches per individual genome. Given the reduced representation focus on *Sbf1* cut sites and size selection step, the resulting blocks are inflated in size. Hence, admixture timing estimates are likely skewed toward more recent timing of admixture events. Following (*64*), we estimated the number of generations since admixture for diploid genomes from the equation *B* = (0.04) * T^*^L^*^z(1 − *z*) where *B* is the estimated number of ancestry switches, *T* is the number of generations since admixture, *L* is the total genome length [2085 cM for autosomes and 111 cM for the X chromosome (*65*)], and *z* is the genome-wide red wolf ancestry proportion specific to autosomes or X chromosome. To convert the generation time into calendar years, we averaged the number of years since admixture across two generation times: the commonly estimated value of 4 years per generation and an estimate of 2 years per generation to account for scenarios in which a fraction of canids breed in their first year of life (*66*, *67*).

#### Morphology and red wolf ancestry

We assessed the relationship between ancestry estimates and body size with mixed effect linear regression models with the lme4 package in program R (*68*). Response variable was body weight (kilograms), given that it is a consistently measured morphometric that encompasses overall body size. We ran separate mixed models with autosomal and X-linked red wolf ancestry estimates as explanatory variables, included sex and age as covariates, and used the geographic region where a coyote was trapped as a random effect to account for nonindependence-associated similarities between trapping regions. We constructed eight a priori candidate models, where the top model was selected on the basis of AIC values. We determined significance of variables in the top models on the basis of 95% confidence intervals not overlapping zero. All models fit a normal distribution. Models were evaluated for fit and adherence to assumptions by visualizing residuals and fitted values.

#### Maximum likelihood clustering method for population genetic structure analysis

We used the program ADMIXTURE (*69*) to assess proportional cluster membership (*Q*) across nine data partitions (*K* = 2 to 10). We implemented the cross-validation (cv) error flag to assess the best-fit partition given the genotype data. Although the lowest cv error is presumed to be the best-fit partition, we surveyed partitions with similar cv errors to evaluate the patterns of clustering with increasing partitions. Cluster patterns are likely influenced by relatedness and inbreeding, often an aspect of capture populations that is unavoidable (i.e., captive red wolf population).

#### Private allele analysis

We used the populations module in STACKS v2 to identify alleles private to the SWLA coyotes with respect to two sample sets that included reference lineages: (i) 10 domestic dogs, 39 coyotes, 19 gray wolves, 10 eastern wolves, and 20 red wolves (10 from the SSP captive population and 10 from NCNEP) genotyped for 41,309 SNPs after filtering for genotypic correlations and HWE; and (ii) 37 coyotes, 9 Texas canids sampled during the early 1970s capture efforts to identify the red wolf founders, and 10 SSP captive red wolves genotyped for 45,994 SNPs after filtering for MAF, missingness, genotype correlation, and deviations from HWE. We additionally conducted a rarefaction method for private allele analysis while controlling for sample size variation in the number of genomes sampled in the program Allelic Diversity Analyzer (ADZE) (*70*). As both analyses were focused on estimating the number of private alleles in the SWLA, we set the parameter *G* of sample size to 100.

## Notes

View/request a protocol for this paper from *Bio-protocol*.
