# Supplementary material for: Reviving ghost alleles: Genetically admixed coyotes along the American Gulf Coast are critical for saving the endangered red wolf
Source: Sci Adv. 2022 Jun 29;8(26):eabn7731. doi: 10.1126/sciadv.abn7731 (PMC9242586; doi:10.1126/sciadv.abn7731)

Science Advances
AAAS

Supplementary Materials for

Reviving ghost alleles: Genetically admixed coyotes along the American Gulf Coast are critical for saving the endangered red wolf

Bridgett M. vonHoldt *et al.*

Corresponding author: Kristin E. Brzeski, kbrzeski@mtu.edu; Bridgett M. vonHoldt, vonholdt@princeton.edu

Sci. Adv. **8**, eabn7731 (2022)
DOI: 10.1126/sciadv.abn7731

The PDF file includes:

Legends for tables S1 and S2
Fig. S1

Other Supplementary Material for this manuscript includes the following:

Tables S1 and S2

Table S1.

Sample information for each genome analyzed, including species, location of sample origin, population identifier, Louisiana Parish, latitude and longitude, sex if known, year of birth, group designation for ancestry analysis (e.g. reference vs. query), per sample proportion of missing data, additional notes, and reference if the sample was previously published.

See file SupplementalTableS1.xlsx

Table S2. Information and morphometric measurements (in centimeters) for 28 coyotes captured and their parish of origin in southwestern Louisiana. Measurements include skull length (SL), skull width (SW), body length (BL), tail length, shoulder height (SH), hind foot (HF), ear, front foot length (FFL), front foot width (FFW), hind foot length (HFL), and hind foot width (HFW).

See file SupplementalTableS2.xlsx

Figure S1. Rarefaction curves for the mean number of private alleles detected per locus per group for varying sample sizes (g). The left panel includes contemporary canids; the right panel includes only coyotes and red wolves to compare to coyotes from southwestern Louisiana and canids captured during the 1970s. (Abbreviations: SWLA, southwestern Louisiana)

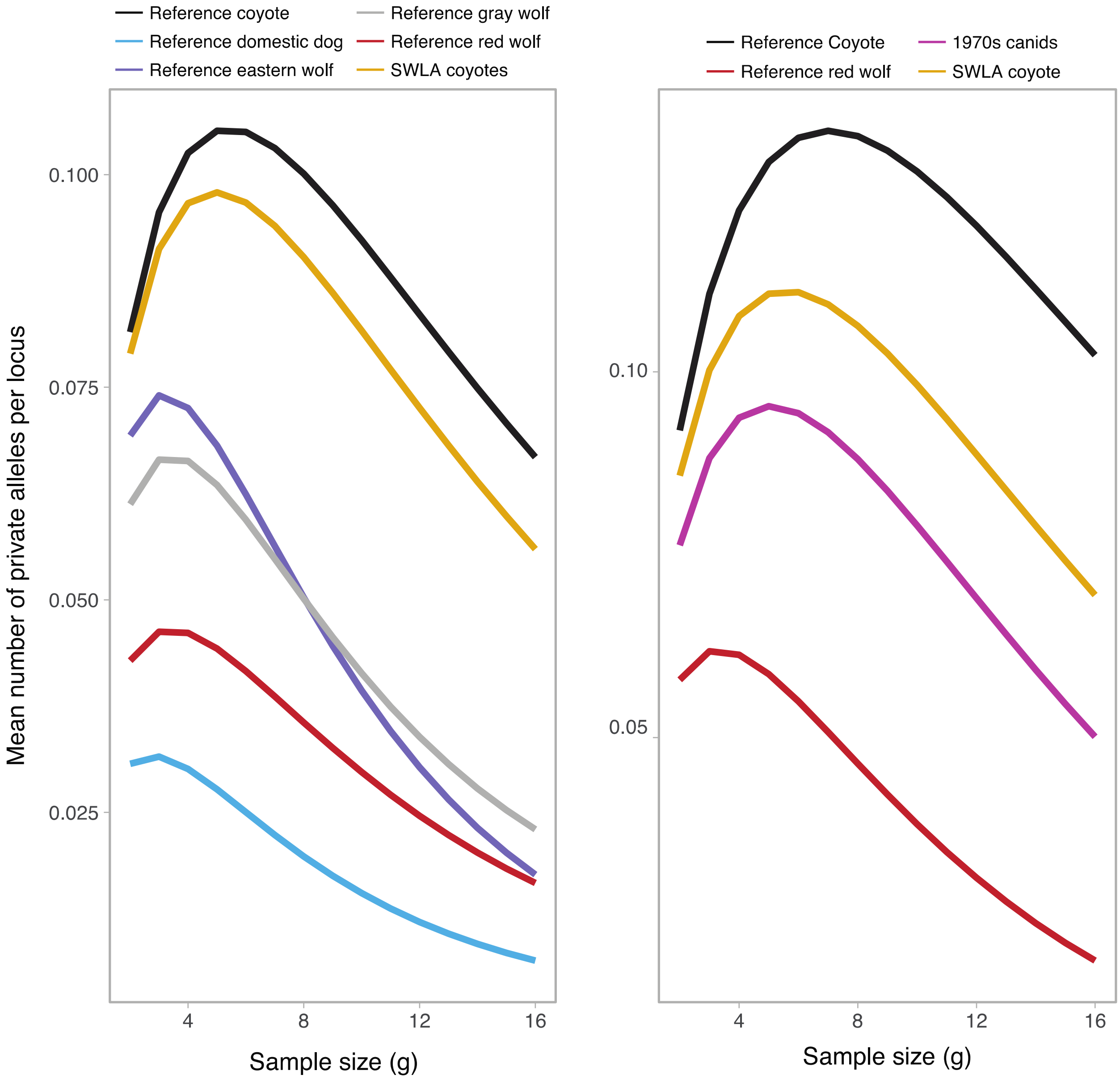

Supplement: Supplementary file 1 — Fig. S1 [file sciadv.abn7731_sm.pdf]
